# Protocol for permanent gene repression by CRISPR-adenine base editing of promoter CCAAT motifs

**DOI:** 10.1016/j.xpro.2025.104075

**Published:** 2025-09-11

**Authors:** Karim Daliri, Kendell Clement

**Affiliations:** 1 Institute for Neurophysiology, Centre for Physiology and Pathophysiology, Medical Faculty and University Hospital of Cologne, University of Cologne, 50931 Cologne, Germany; 2 Department of Biomedical Informatics, University of Utah, Salt Lake City, UT, USA

**Keywords:** Cell Biology, Health Sciences, Genetics, Genomics, Sequencing, Molecular Biology, Gene Expression, CRISPR

## Abstract

Here, we present a protocol to achieve permanent downregulation of gene expression by editing the CCAAT box in promoter regions using CRISPR-adenine base editors (ABEs). We outline steps for guide RNA (gRNA) design, transfection, genomic DNA extraction, Sanger sequencing, and gene expression quantification. The protocol is optimized for mammalian cell lines (e.g., NIH3T3). It allows for precise disruption of transcription factor binding site without double-strand breaks and offers a novel alternative to RNAi or CRISPR interference (CRISPRi).

For complete details on the use and execution of this protocol, please refer to Daliri et al.^1^

## Highlights

• Instructions for selecting sgRNAs and optimal ABEs to target promoter motifs
• Steps for Sanger sequencing of CCAAT box inside promoter wild and edited site
• Guidance on transfection, clonal expansion, and gene repression quantification in NIH3T3

## Abstract (editor highlights)

Publisher’s note: Undertaking any experimental protocol requires adherence to local institutional guidelines for laboratory safety and ethics.

## Graphical abstract

**Figure:**
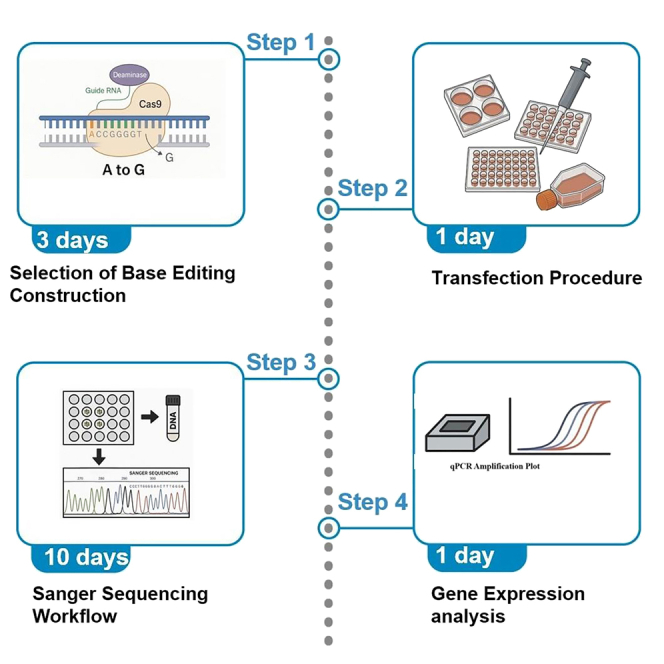

## Before you begin

### Innovation

Traditional gene repression methods such as RNAi, shRNA, or CRISPRi often rely on transient expression systems, lack precision, and are limited by variability in silencing efficiency. To address these limitations, we developed a base-editing approach that targets highly conserved promoter motifs—specifically the CCAAT box—to achieve irreversible transcriptional silencing. This protocol utilizes a Cas9-derived adenine base editor (ABE8e), which enables A-to-G conversions without introducing double-strand breaks or requiring donor templates. By integrating motif-specific guide RNA design with an optimized, selection-free workflow, this protocol allows researchers to precisely modulate gene expression at the epigenetic interface of promoter function. A key innovation is the motif-based targeting logic, which focuses on functionally validated transcription factor binding elements rather than arbitrary upstream regions. Additionally, the protocol is designed to work in multiple mammalian cell types, making it adaptable and scalable. The method preserves genome integrity, eliminates the need for clonal selection, and is particularly valuable in systems where long-term repression is required without persistent transgene expression. The simplicity, durability, and reproducibility of this base-editing framework open new avenues in studying transcriptional regulation, validating disease-associated regulatory elements, and developing preclinical gene-silencing strategies for hard-to-drug targets. While most gene editing strategies focus on coding regions to disrupt gene function, this protocol introduces a regulatory-centric paradigm by editing non-coding promoter elements. Promoter regions often contain multiple overlapping transcription factor binding sites; among them, the CCAAT motif is one of the most conserved and widely used. By selectively editing adenines within this motif, the protocol enables stable suppression of gene transcription without affecting the downstream coding sequence. This mechanism avoids complications such as frameshift mutations or nonsense-mediated decay and instead simulates a naturally repressed transcription state. Such a strategy is highly relevant for disease modeling in contexts like fibrosis, cancer, and neurodegeneration—where pathological gene expression often stems from regulatory dysregulation rather than coding mutations. In preclinical research, this protocol can be used to investigate the functional importance of non-coding regulatory elements in gene networks. Furthermore, it lays the groundwork for therapeutic gene repression in scenarios where long-term silencing of disease-driving genes is desired—without the risks associated with genome fragmentation or off-target effects of RNAi. For example, sustained downregulation of extracellular matrix genes in fibrotic disorders, metabolic regulators in obesity or diabetes, or aberrant transcription factors in cancers could be achieved by promoter editing. In the future, with further refinement and delivery improvements (e.g., via AAV or LNPs), this platform may contribute to precision epigenetic therapies that act at the level of transcriptional initiation—offering a novel layer of control in gene therapy design.

**Figure 1:**
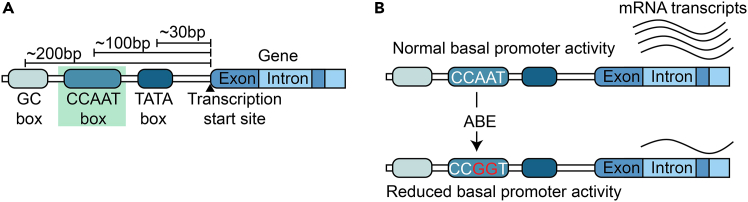
Schematic of promoter architecture and adenine base editing strategy (A) Schematic showing common promoter elements: GC box (∼200 bp upstream), CCAAT box (∼100 bp), and TATA box (∼30 bp) relative to the transcription start site. (B) Adenine base editing (ABE) converts CCAAT to CCGGT in the CCAAT box, diminishing basal promoter activity, leading to reduced mRNA output.

Promoter Modulation by Base Editing (PMBE) is a targeted strategy that allows for precise and permanent regulation of gene expression by editing cis-regulatory elements—such as the CCAAT box—within promoter regions^1^ (Figure 1)⁠. This approach leverages CRISPR-Cas9 adenine base editors (ABEs) to convert adenine to guanine without inducing double-strand breaks or requiring donor DNA templates.^2^ By disrupting key promoter motifs, PMBE achieves sustained downregulation of gene transcription. Unlike transient gene regulation techniques such as RNA interference (RNAi)^3^⁠ or CRISPR interference (CRISPRi),^4^ PMBE introduces irreversible nucleotide changes that result in stable modulation of gene activity. PMBE has the potential for broad applications in functional genomics, disease modeling, and therapeutic development, including potential use in modulating the expression of oncogenes, neurodegeneration-associated genes, or metabolic regulators. Its ability to achieve durable gene repression through minimal genomic intervention makes PMBE a powerful alternative to conventional gene knockdown tools.

## Key resources table

**Table:** 

REAGENT or RESOURCE	SOURCE	IDENTIFIER
**Chemicals, peptides, and recombinant proteins**		

Cell culture medium (DMEM)	Gibco	Cat#11965092
Fetal bovine serum (FBS)	Gibco	Cat#10270106
Penicillin/streptomycin	Gibco	Cat#15140122
PBS (phosphate-buffered saline)	Gibco	Cat#10010023
Tris-acetate-EDTA (TAE)	Carl Roth	Cat#CL86.1
Agarose gel	Invitrogen	Cat#16500-500
TrypLE	Thermo Fisher Scientific	Cat#12605010
Opti-MEM medium	Thermo Fisher Scientific	Cat#31985070
Transfection reagent (Lipofectamine 3000)	Invitrogen	Cat#L3000008
TransIT-mRNA	Mirus Bio	Cat#MIR2250
Gel loading dye, purple (6X)	NEB	Cat#B7025S
DNA ladder	NEB	Cat#N3231S
peqGREEN DNA/RNA dye	Avantor	Cat#7323196
Trypan blue stain 0.4%	Invitrogen	Cat#15250061
Proteinase K	NEB	Cat#P8107S
Dimethyl sulfoxide (DMSO)	AppliChem	Cat#2045215
DNase/RNase-free distilled water	Invitrogen	Cat#10977035 RRRRRRRR

**Experimental models: Cell lines**		

NIH3T3 (mouse fibroblast cell line)	ATCC	Cat# CRL1658

**Critical commercial assays**		

cDNA synthesis kit	Invitrogen	Cat#15596018
RNeasy mini kit	QIAGEN	Cat#74004
DNeasy blood & tissue kit	QIAGEN	Cat#69581
OneTaq DNA polymerase	NEB	Cat#M0480S
PowerUp SYBR Green master mix	ABI	Cat#A25742
PureLink PCR purification kit	Invitrogen	Cat#K310001

**Recombinant DNA**		

gRNA expression vector Addgene ID: 132777		
gRNA expression vector	Addgene	ID: 132777

**Oligonucleotides**		

sgRNA for Col1a1 promoter	Thermo Fisher Scientific	Sequence: 5′-CCCCAATTTGGAACGAAGAG-3′
qPCR primer: GAPDH forward	Thermo Fisher Scientific	Daliri et al.^1^
qPCR primer: GAPDH reverse	Thermo Fisher Scientific	Daliri et al.^1^
qPCR primer: Col1a1 forward	Thermo Fisher Scientific	Daliri et al.^1^
qPCR primer: Col1a1 reverse	Thermo Fisher Scientific	Daliri et al.^1^
Promoter PCR primer: forward	Thermo Fisher Scientific	Daliri et al.^1^
Promoter PCR primer: reverse	Thermo Fisher Scientific	Daliri et al.^1^

**Software and algorithms**		

ImageJ (for gel quantification)	NIH	https://imagej.nih.gov/ij/
CCtop (CRISPR target prediction)	Heidelberg University	https://cctop.cos.uni-heidelberg.de/
CHOPCHOP (v.2.0)	University of Bergen	https://chopchop.cbu.uib.no
EditR (v.1.0)	Moriarity Lab	http://baseeditr.com/
Primer-BLAST	NCBI	https://www.ncbi.nlm.nih.gov/tools/primer-blast/
GraphPad Prism (v.9)	GraphPad	https://www.graphpad.com/
Applied Biosystems 7500 software	Thermo Fisher Scientific	https://www.thermofisher.com/order/catalog/product/ 4351104

**Other**		

Filter tips, 0.1–10 μL	VWR	Cat#76322-132
Filter tips, 2–200 μL	VWR	Cat#76322-134
Filter tips, 100–1,000 μL	VWR	Cat#76322-156
Standard microcentrifuge tubes, 1.5 mL	Eppendorf	Cat#0030 125.150
Serological pipette	VWR	Cat#53498-105
P200 micropipette	Gilson	Cat# F123602
NanoDrop 2000 spectrophotometer	Thermo Fisher Scientific	Cat# ND-2000
Light microscope	Nikon	Eclipse TS100
Filter flasks	VWR	Cat#10040-436
Shaking incubator (37°C, 220 rpm)	Thermo Fisher Scientific	Cat#SHAK-22037
Tissue culture plate, 6 wells	BD Falcon	Cat#353046
Tissue culture plate, 24 wells	BD Falcon	Cat#353047
Tissue culture plate, 96 wells	BD Falcon	Cat#353075

## Materials and equipment

**Table:** Components for agarose gel electrophoresis

Component	Amount
PCR product	5 μL
6× loading dye	1 μL
Agarose	2%
TAE buffer	1×
peqGREEN dye	1×

**CRITICAL:** Agarose (when molten): Causes burns on contact. Handle hot solutions with heat-resistant gloves.

***Note:*** peqGREEN dye and Loading dye: Store at −20°C (long term) or 4°C (short term, up to 1 month) protected from light.

**Table:** Lysis buffer composition for DNA extraction (for 1 L preparation)

Reagent	Final concentration	Amount
Tris-HCl (pH 7.5)	10 mM	10 mL of 1 M stock
SDS (10%, wt/vol)	0.5%	5 mL of 10% stock
Nuclease-free water		up to 1,000 mL

**CRITICAL:** SDS (Sodium dodecyl sulfate): Harmful if inhaled, ingested, or absorbed through the skin. Causes skin and eye irritation. Always wear gloves, lab coat, and safety goggles when handling. Work in a fume hood when preparing stock solutions to avoid inhalation of powder or aerosol.

***Note:*** The lysis buffer can be stored at 25°C, up to 6 months.

## Step-by-step method details

### Sanger sequencing of promoter in wild-type cells

**Timing: 2 days**

**Figure 2:**
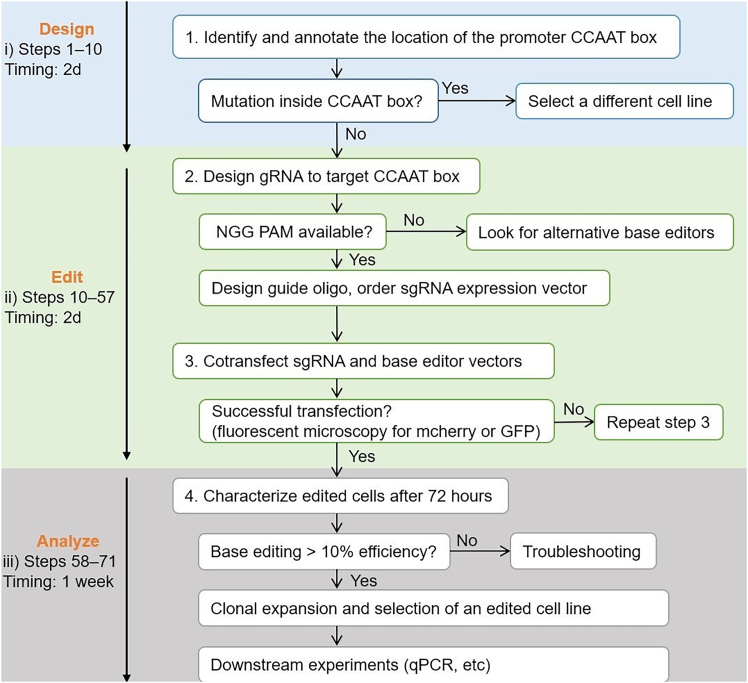
The generation of isogenic cell lines by the PMBE approach consists of three stages: (i) Identify the location and the sequence of CCAAT (steps 1–10, blue boxes), (ii) design gRNA to target the promoter CCAAT box and perform editing experiments (steps 10–57, green boxes), and (iii) gene expression evaluation (e.g., using qPCR) (steps 58–71, gray boxes)

This section describes how to amplify and sequence the promoter region of the target gene in wild type cells. Confirming the sequence context is essential for accurate sgRNA design and successful base editing of CCAAT motifs (Figure 2).

1. To identify the promoter region of the gene of interest, first locate the transcription start site (TSS) using the Eukaryotic Promoter Database (EPD: https://epd.epfl.ch/), which provides curated promoter annotations for many eukaryotic species.
2. Select a promoter region approximately −200 to +100 bp, ensuring that it includes at least one CCAAT box.

***Note:*** In some genes, multiple or redundant CCAAT boxes may exist—preferably select one within the −75 to −85 bp range relative to the TSS. Promoters lacking a CCAAT box (e.g., TATA-less promoters) should be noted, as editing efficiency and relevance may be lower.

3. Use the “Search Motif” tool in EPD (https://epd.epfl.ch/motif_search.php) or manually scan the sequence for the CCAAT consensus sequence within the promoter.
4. Choose a 300–400 bp region that contains the CCAAT box as centrally as possible.
5. Design primers for this region using Primer-BLAST (https://www.ncbi.nlm.nih.gov/tools/primer-blast).

**Pause point:** Place the expected base edit site near the center of the amplicon.

***Note:*** In genes with multiple CCAAT boxes, consider designing more than one sgRNA targeting different boxes, if necessary.

6. Order the oligonucleotide primers from a commercial provider (e.g., IDT: https://www.idtdna.com/) with standard purification (desalted).
7. Dilute the Fwd and Rev-designed primers to a final concentration of 10 μM.

**Pause point:** Dilute 10 μl primer stock with 90 μl ddH_2_O; store at −20°C.

8. Extract DNA from a cell pellet using the DNA extraction kit, following the manufacturer’s instructions.
9. Set up a master-mix for all the PCR products in a 0.5 ml microcentrifuge tube for each reaction.
10. Conduct PCR on a thermocycler using the OneTaq Hot Start Master Mix with Standard Buffer (NEB, Cat#M0486) and the reaction conditions specified in Tables 1 and 2.

**Table 1:** PCR cycling conditions

Steps	Temperature	Time	Cycles
Initial Denaturation	95°C	30 sec	1
Denaturation	95°C	3 min	22–35 cycles
Annealing	55°C	30 sec	
Extension	72°C	4 min	
Final extension	72°C	1 min	1
Hold		8°C	forever

**Table 2:** PCR cycling conditions

Reagent	Amount (μL)
DNA template	3
DNA Polymerase	12.5
Primer 1	0.5
Primer 2	0.5
ddH_2_O	8.5
Total	25

**CRITICAL:** The optimal annealing temperature must be determined experimentally for each primer pair and target site.

11. Analyze PCR products by agarose gel electrophoresis as follows:

**CRITICAL:** Run electrophoresis at 120 V for 45 minutes. You should see a single sharp band of the expected size (300–500 bp).

12. Purify the PCR product.

### sgRNA design and selection of base editor variants

**Timing: 1 week**

This section provides instructions for designing sgRNA and selecting the appropriate adenine base editor (ABE) variant for efficient and specific targeting of CCAAT motifs in the promoter region.

13. Determine which targeted base conversion is necessary for your experiment.
14. For CCAAT motif targeting, use ABEs for A:T-to-G:C or T-to-C conversions.
15. Select a high-activity base editor, such as ABE8 or ABE8e, to maximize editing efficiency.

***Note:*** Synthetic mRNA encoding ABE8e were synthesized in the laboratory of Prof. David R. Liu (Broad Institute, Harvard University).

16. Design guide RNAs for mutagenesis using CHOPCHOP v.2 (https://chopchop.cbu.uib.no/).
17. Select target sites adjacent to an NGG PAM (SpCas9) with a spacer length of 20 nucleotides.
18. Prioritize sgRNAs that position the target adenine centrally within the editing window.

***Note:*** To ensure optimal editing efficiency with Cas9-ABE8e, select a target adenine located within **positions 4 to 9 nucleotides of the target sequence**. This range has been identified as the canonical editing window for ABE8e.^5^ Among these, **positions 5–6** are often reported to yield the highest editing activity.

19. Select the sgRNA expression vector.

**Pause point:** The bacterial sgRNA expression vector (Addgene ID: 132777) is available from Addgene.

20. Clone the sgRNA oligonucleotides into the sgRNA plasmid to target the desired genomic loci.

***Note:*** To save time, consider ordering pre-cloned sgRNA constructs from a preferred supplier (e.g., GenScript).

### Targeted gene repression in NIH3T3 cells

**Timing: 4 days**

This section details the process for culturing and preparing NIH3T3 cells to achieve optimal confluency and health prior to transfection, ensuring efficient delivery of gene editing components and successful targeted gene repression.

21. Culture NIH3T3 cells in a 6-cm dish using high-glucose DMEM.
22. Culture NIH3T3 cells in high-glucose DMEM.
23. Seed cells one day prior to transfection to achieve 70%–80% confluency on the day of transfection (18–24 hours before transfection), as follows:
  a. Remove the culture medium from the dish.
  b. Gently wash the cells with 1× PBS buffer.
  c. Add fresh growth medium and adjust cell density as needed.
24. Add 2 ml of TrypLE and incubate at 37°C for 5 minutes.
25. Add 4 ml of freshly warmed DMEM.
26. Mix thoroughly by pipetting up and down to ensure the cells are completely dissociated.

**CRITICAL:** Avoid growing cells beyond 80% confluency or using them beyond passage 15.

27. Plate NIH3T3 cells for transfection as follows:
  a. Seed cells in 12- or 24-well plates 16–24 hours before transfection.
  b. Adjust the cell density to 0.8 × 10^5^ cells/mL per well.
  c. Add a total volume of 500 μL growth medium per well.

### Delivery of the sgRNA and base editor

**Timing: 1 day**

This section describes the preparation and delivery of sgRNA and adenine base editor components into NIH3T3 cells to facilitate efficient gene editing. Proper handling and timing of reagent mixing are essential for optimal transfection efficiency.

28. Use a microscope to ensure the cells are 70%–80% confluent.
29. Replace DMEM medium in wells with fresh DMEM and place cells back in the incubator.
30. Warm transfection materials such as optiMEM and plasmids to 25°C 15 min before starting.
31. Prepare volumes as described below (per well of a 24-well plate, see Table 3).

**Table 3:** Transfection composition (24-well plate)

Component	Amount	Volume
OptiMEM		100 μL
Boost Reagent		2 μL
TransIT Solution		2 μL
sgRNA plasmid	300 ng/μL	1 μL
Adenine Base editor	900 ng/μL	1 μL
GFP vector	10 ng/μL	1 μL
Total per well		50 μ
32. Mix thoroughly with a pipette and incubate for 5 minutes at 25°C.

**CRITICAL:** Exceeding 5 minutes of incubation is not recommended, as it could potentially decrease the efficacy of the transfection process.

***Note:*** This indicates the required vector quantities per well for 24-well plates. For other plate sizes, adjust the masses of all volumes proportionally.

33. Add 50 μl to each well of the plate, gently swirl, and return the plates to the incubator.

**CRITICAL:** Perform transfections in triplicate and include appropriate controls (GFP or mCherry) for accuracy.

34. Assess plates by observing GFP or mCherry expression using a fluorescence microscope after 24 hours.

**Pause point:** Typically, more than 70% of cells are transfected.

35. Aspirate and replace with fresh DMEM to reduce lipid toxicity.
36. Incubate the cells for 48–72 hours before passaging for subsequent applications.

### Preparation of cells for Sanger sequencing

**Timing: 1 day**

This section outlines the preparation of cell lysates for gDNA extraction suitable for Sanger sequencing, including an efficient lysis buffer recipe for small-scale samples.

***Note:*** For extracting DNA from a small number of cells, you can use e lysis buffer for sequencing applications. DNA extraction with this solution involves simple cell lysis without the need for subsequent purification.

37. Mix proteinase K (NEB) into the cell lysis buffer at a dilution ratio of 1:1,000 (vol/vol).

**CRITICAL:** Store proteinase K at −20°C for up to 12 months.

38. Add lysis buffer directly to plates from Step 30.

**Pause point:** In general, 150 μl of lysis buffer is sufficient for each well of a 24-well plate.

39. Incubate the plates at 37°C for 1 hour.
40. Transfer the lysis mixture to PCR strips and deactivate the proteinase K by heating at 80°C for 20 minutes.

**CRITICAL:** The DNA can be temporarily stored at 8°C for two weeks or frozen at −80°C for months.

41. Assess the quality and quantity of the extracted DNA using a NanoDrop spectrophotometer.

***Note:*** The concentration of the isolated DNA of 50–100 ng/*μ*l indicates an acceptable yield.

**CRITICAL:** The absorbance ratios (260/280 nm) should range from 1.8 to 2.2.

42. Perform Sanger sequencing of the product using the same sequencing primers (Keep diluted primers in −20°C for 6 months) used to sequence the genomic target site (Steps 1–10) in unedited NIH3T3.
43. Determine base editing efficiencies from Sanger sequence chromatograms using EditR.

### Isolation of clonal cell lines by dilution approach

**Timing: 10 days**

This section describes the steps for isolating single-cell-derived clonal lines from edited cell populations by limiting dilution, enabling downstream analysis of genetically uniform clones.

44. Dissociate the cells from the transfected wells 48 hours after transfection as follows:
  a. Add Trypsin-EDTA (0.05%) to the wells.
  b. Incubate the cells at 37°C for 3–5 minutes until detachment is observed.
  c. Gently pipette the cells to ensure complete dissociation.
  d. Pass the cell suspension through the mesh cap of a cell strainer tube to obtain a single-cell suspension.

**CRITICAL:** Examine the dissociated cells under a microscope to confirm successful dissociation.

45. Count the cells in each 24-well plate and perform serial dilutions in DMEM medium to achieve a final concentration of 0.5 cells per 100 μl,

***Note:*** To minimize the presence of multiple cells per well. It is suggested to use 70 cells in 12 ml for each 96-well plate and to plate at least three 96-well plates for each transfected population.

46. Using a multichannel pipette, dispense 100 μl of the diluted cells into each well of a 96-well plate and

**Pause point:** Place the 24-well plate in the CO_2_ incubator at 37°C.

47. Inspect the colonies for a clonal appearance approximately one week after plating.
48. Return the cells to the incubator.
49. Allow cells to expand for about 2 weeks.
50. After 2 weeks, passage each colony into a new 96-well plate by duplicate (1:2).
51. After 3–4 days use one well to extract DNA.
52. Determine the sequence at the target site by PCR and Sanger sequencing as described in Steps 4–9.
53. Confirm the presence of the desired mutation in the genotype of the new cell lines.
54. Determine the sequences at these off-target sites through PCR and Sanger sequencing, as detailed in Steps 1–9.

***Note:*** Examine clonal lines for editing at off-target loci. For each locus, we recommend Cas-OFFinder with default parameters for S. pyogenes Cas9 against the species reference sequence to identify the top three predicted off-target sites.

### Cryopreservation of PMBE-edited cell lines

**Timing: 1 h**

This section details the procedures for freezing and storing PMBE-edited cell lines to ensure long-term preservation and viability of gene-edited populations for future experiments.

***Note:*** Once the cells have reached approximately 80% confluence, they are ready for cryopreservation.

55. Remove the medium from the wells designated for banking and add 1 mL of Trypsin.
56. Incubate the plates at 37°C for 5 minutes.
57. Use a P200 micropipette to rinse the wells and collect the cells.
58. Transfer the cell suspension to a 15 mL conical flask and add an equal volume of DMEM medium.
59. Centrifuge the mixture at 200 × g for 5 minutes.
60. Aspirate and discard the supernatant without disturbing the cell pellet.
61. Resuspend the cell pellet in a freezing medium (90% FBS and 10% DMSO at a density of 1 × 10^6^ cells/ml).
62. Label the cryovials and transfer 1 ml of the cell suspension into each cryovial.
63. Place the cryovials in a Mr. Frostie container and immediately transfer them to a −80°C freezer.
64. The following day, transfer the cryovials to liquid nitrogen storage for long-term preservation.

### Specific gene downregulation in edited cells

**Timing: 1 day**

This section describes how to assess specific gene downregulation in edited cells by performing relative qPCR analysis to compare gene expression levels between edited and non-edited samples.

**CRITICAL:** Use a relative qPCR quantification method to quantify the level of the repressed gene of interest in edited cells compared to non-edited cells.

65. Use primer Database: Search database like RTPrimerDB (http://medgen.ugent.be/rtprimerdb) for pre-validated qPCR primer sets targeting your gene of interest.
66. Input the target nucleotide sequence into a BLASTn search (http://www.ncbi.nlm.nih.gov/blast) using the default parameters.
67. Order primers from a supplier.

**Pause point:** Ideally, three different sets of oligonucleotides should be ordered and tested.

### qPCR experiments

**Timing: 4 h**

This section outlines the steps for conducting qPCR experiments to assess gene expression changes, including RNA extraction, cDNA synthesis, and qPCR setup in edited and control samples.

**CRITICAL:** RNA is sensitive to temperature; therefore, avoid repeated thawing and freezing.

68. Extract RNA from cells and quantify RNA using a NanoDrop spectrophotometer.

***Note:*** An OD260/280 ratio of 1.8–2.0 signifies high- quality RNA.

69. Determine RNA integrity using 1% agarose gel electrophoresis.

**CRITICAL:** Store RNA on ice during handling; store at −80°C for up to 12 months.

**Pause point:** The presence of two distinct bands for the large and small subunit ribosomal RNAs (rRNA), with the larger band being about twice as intense as the smaller one, indicates intact RNA.

70. Use 1 μg of RNA as the template for synthesizing complementary DNA (cDNA)).

**Pause point:** The cDNA can be stored at −20°C for up to 6 months.

71. Dilute cDNA to a total of 100 μl with ddH_2_O for qPCR experiments.
72. Use 1 μl of the cDNA for a 10 μl PCR amplification.

**CRITICAL:** Use stable reference genes (e.g., GAPDH) for qPCR data normalization.

73. Make a qPCR mastermix by adding the reagents in the order shown in the table 4.

**Table 4:** SYBR qPCR mix composition

Reagent	Amount (μL)
cDNA template	3
PowerUp SYBR Green	5
Primer 1	0.67
Primer 2	0.67
ddH_2_O	0.66
Total reaction	10

***Note:*** Perform the reactions in duplicate.

**Figure 3:**
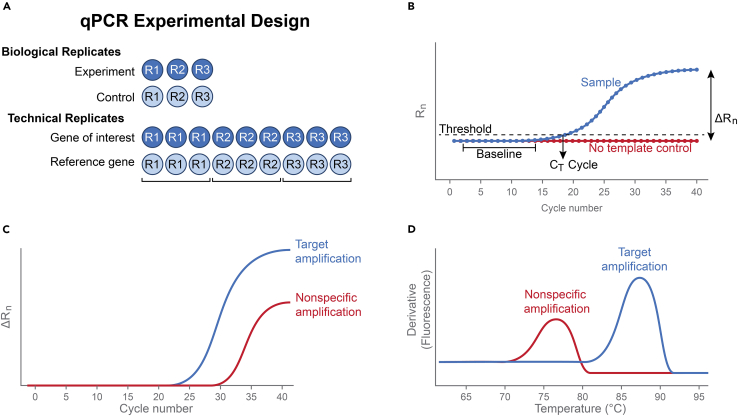
Quantitative PCR (qPCR) analysis using SYBR Green I dye chemistry (A) Experimental design showing biological (R1, R2, R3) and technical replicates for both gene of interest and reference gene. (B) Representative amplification plot illustrating baseline, threshold (Ct), and ΔRn. (C) Amplification curves comparing specific target amplification with nonspecific amplification in the no-template control (NTC). (D) Dissociation curve demonstrating that nonspecific products in NTC wells have a distinct melting temperature compared to the specific amplicon.

***Note:*** The exact cycling conditions will vary based on the enzyme and instrument used. The following conditions are generally accepted for conventional block systems and are suitable for the Applied Biosystems Fast 7500 Real-Time PCR System (Table 5).

74. Add 10 μl of master mix to each reaction tube,
75. Gently mix by pipetting up and down several times without creating bubbles.
76. Centrifuge the tubes at 200 × g for 10 seconds.
77. Run in qPCR instrument using a three-step protocol (Figure 3).

**Table 5:** qPCR cycling condition

Steps	Temperature	Time	Cycles
Initial Denaturation	95°C	5 min	1
Denaturation	95°C	10 sec	40 cycles
Annealing	60°C	30 sec	
Extension	60°C	30 sec	
Final extension	60°C	1 min	1
Hold	4°C	forever	

### Data analysis and visualization

**Timing: 1 h**

This section describes how to analyze and visualize qPCR data, including assessment of PCR specificity, use of real-time PCR analysis software, and interpretation of gene expression results.

**CRITICAL:** To ensure reaction specificity, the PCR product should be analyzed. A melt curve analysis at the end of the PCR cycles will confirm primer annealing specificity, indicated by a single sharp peak (Figure 3B).

78. Analyze data using real-time PCR analysis software.

**CRITICAL:** All qPCR instruments come with data analysis software, and the 2^–ΔΔCT^ method.

## Expected outcomes

**Figure 4:**
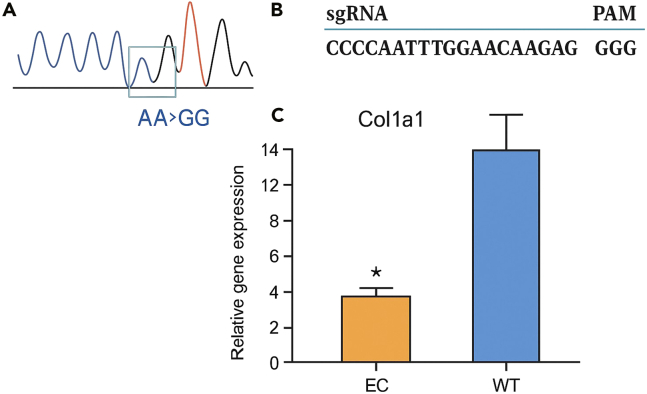
Efficient promoter base editing downregulates Col1a1 expression (A) Sanger sequencing chromatogram confirming precise adenine base editing at the −98 position within the Col1a1 promoter (AA>GG). (B) sgRNA target sequence and adjacent PAM motif (GGG) used for CRISPR-Adenine Base Editing (ABE) of the Col1a1 promoter. (C) Quantitative RT-PCR analysis reveals significantly reduced Col1a1 mRNA levels in edited clones (EC) compared to wild-type (WT) fibroblasts. Data are presented as mean ± SEM; ∗P < 0.05 by unpaired two-tailed t-test.

This protocol can be applied to achieve permanent gene expression modification in mammalian cells by targeting adenine base editing to the CCAAT box within the promoter of the gene of interest. The CCAAT box plays a crucial role in the gene expression of cells, and mutations in the CCAAT motif cause a several-fold decrease in transcription activity both in vitro and in vivo. The CCAAT box is crucial for the binding of transcription factors that facilitate the initiation of transcription. When mutations occur in this region, the binding affinity of these transcription factors may be diminished, leading to a decrease in transcriptional activity and, consequently, lower levels of mRNA production. This reduction in mRNA expression can be accurately quantified using quantitative PCR (qPCR). By comparing the qPCR results of the mutated CCAAT box with those of the unedited, wild-type sequence, researchers can measure the extent of transcriptional repression caused by the mutation. This comparative analysis can be used to understand the functional impact of specific mutations on gene expression. Due to the robustness of base editing technology, multiple targeting and delivery strategies can be employed, increasing the likelihood of achieving the desired editing properties for a specific application. In an amenable cell line, such as NIH3T3, a minimum editing rate of 15% in bulk-sorted populations should be achieved. If editing rates are lower, it is not recommended to proceed with single-cell clonal expansion. In cases of low editing efficiencies in bulk-sorted or clonal cell populations, it is advised to optimize the transfection protocol by adjusting the total amount of DNA delivered and the sgRNA-to-BE ratios. Additionally, users may need to evaluate alternative sgRNA sequences or base editors to enhance targeting levels. In this protocol, we targeted the *Col1a1* promoter using a validated sgRNA in NIH3T3 cells, achieving approximately 18% editing efficiency as confirmed by Sanger sequencing. This gene was chosen because *COL1A1* encodes the most abundant structural protein in the human body, and NIH3T3 fibroblasts are an established model for collagen-related studies.^1^ Sanger sequencing and RT-qPCR analysis confirm successful base editing of the Col1a1 promoter and a marked reduction in Col1a1 mRNA levels (Figure 4).

## Quantification and statistical analysis

All qPCR data were analyzed using Microsoft Excel and GraphPad Prism software (GraphPad, https://www.graphpad.com/). Gene expression levels were normalized to GAPDH and calculated using the ΔΔCt method. Statistical comparisons between groups were performed using unpaired two-tailed t-tests in GraphPad Prism. Data should be presented as mean ± standard deviation (SD) unless otherwise specified. A p-value < 0.05 are considered statistically significant.

## Limitations

While Promoter Modulation by Base Editing (PMBE) offers a precise and permanent approach to modulate gene expression, it does have certain limitations. One significant limitation is the dependency on the presence of a protospacer adjacent motif sites within promoter regions.^6^^,^^7^ This limitation can hinder the ability to target specific regulatory elements if the necessary PAM sequence is not present. Additionally, the editing window of base editors is relatively narrow, typically spanning positions 4 through 8 within the protospacer, which can limit the flexibility in targeting specific bases within promoters.^8^^,^^9^ Furthermore, unintended edits, such as bystander edits within the same protospacer or off-target (PAM) sequence, which is required for the CRISPR-Cas9 system to recognize and bind to the target DNA.^10^^,^^11^ The most commonly used Cas9 from Streptococcus pyogenes requires an NGG PAM sequence, which can restrict the availability of target hits (or sites) at other genomic loci, remain a concern.^8^^,^^12^ These unintended edits can result from the deaminase activity affecting multiple accessible adenines within the editing window, leading to undesired mutations. Additionally, the efficiency of base editing can be influenced by the local chromatin environment and DNA accessibility, which may vary between different genomic contexts, potentially affecting the consistency and reliability of CRISPR-based methods.^13^^,^^14^ The long-term effects and potential unintended consequences of permanent promoter modifications also need to be thoroughly evaluated to ensure safety and efficacy in therapeutic applications.^15^^,^^16^

## Troubleshooting

### Problem 1

Non-specific PCR products may result from poor primer design or suboptimal PCR conditions (related to Step: 11).

### Potential solution

Design primers carefully. Perform melt curve analysis to check for specific products.

### Problem 2

No base for conversion in the window (No PAM) so genomics sequence not optimal (related to Step: 17).

### Potential solution

Check minus strand for editable guide sequence.

### Problem 3

Low transfection efficiency can result from suboptimal transfection conditions, poor DNA quality, issues with the cell line, improper incubation conditions, or high cell confluency (above 85%) (related to Step: 27).

### Potential solution

Optimize conditions. Use high-quality DNA. Choose a suitable cell line. Use efficient reagents. Ensure proper incubation. Ensure that cells are less than 85% confluent before transfection.

### Problem 4

Sanger sequencing shows noisy or mixed peaks at the editing site (related to Step: 43).

### Potential solution

Clone PCR products into plasmid vectors followed by individual colony sequencing to distinguish edited from unedited alleles. Use analysis tools such as EditR or TIDE for accurate quantification of editing efficiency. Optimize transfection and editing conditions to increase editing rates before sequencing.

### Problem 5

No effect on gene expression may be due to unreliable gene expression data, such as poor RNA quality or quantity, use of an inappropriate reference gene, or misinterpretation of the data (related to Step: 71).

### Potential solution

Ensure the experimental setup includes appropriate controls (e.g., untreated cells, vehicle controls) for comparison with treated samples. Confirm that the cells remain healthy and viable throughout the experiment. Carefully analyze and interpret gene expression data, taking into account statistical significance and biological relevance. Verify that the data normalization methods are appropriate.

## Resource availability

### Lead contact

Further information and requests for resources and reagents should be directed to and will be fulfilled by the lead contact, Karim Daliri (karimdaliri@gmail.com).

### Technical contact

Technical questions on executing this protocol should be directed to and will be answered by the technical contact, Karim Daliri (karimdaliri@gmail.com).

### Materials availability

This protocol did not generate new materials.

### Data and code availability

This protocol did not generate/analyze new datasets or code.

## Acknowledgments

We gratefully acknowledge the financial support of the Neurophysiology Institute at the Faculty of Medicine, University of Cologne, Germany, for providing technical assistance and access to research facilities during the course of this project.

## Author contributions

K.D. conceived, initiated, and led the project; designed and conducted the experiments; analyzed and interpreted the data; and wrote the first draft of the manuscript. K.C. contributed to data analysis, prepared the figures and tables, and reviewed the manuscript. All authors read and approved the final version of the manuscript.

## Declaration of interests

The authors declare a patent application related to this work.
